# Structural Features and Binding Modes of Thioether-Cyclized Peptide Ligands

**DOI:** 10.3390/biomedicines6040116

**Published:** 2018-12-13

**Authors:** Manuel E. Otero-Ramirez, Toby Passioura, Hiroaki Suga

**Affiliations:** 1 Department of Chemistry, Graduate School of Science, The University of Tokyo, 7-3-1 Hongo, 113-0033 Tokyo, Bunkyo-ku, Japan; motero@chem.s.u-tokyo.ac.jp (M.E.O.-R.); toby@chem.s.u-tokyo.ac.jp (T.P.); 2 JST CREST, The University of Tokyo, 7-3-1 Hongo, 113-0033 Tokyo, Bunkyo-ku, Japan

**Keywords:** macrocyclic, peptide, crystallography

## Abstract

Macrocyclic peptides are an emerging class of bioactive compounds for therapeutic use. In part, this is because they are capable of high potency and excellent target affinity and selectivity. Over the last decade, several biochemical techniques have been developed for the identification of bioactive macrocyclic peptides, allowing for the rapid isolation of high affinity ligands to a target of interest. A common feature of these techniques is a general reliance on thioether formation to effect macrocyclization. Increasingly, the compounds identified using these approaches have been subjected to x-ray crystallographic analysis bound to their respective targets, providing detailed structural information about their conformation and mechanism of target binding. The present review provides an overview of the target bound thioether-closed macrocyclic peptide structures that have been obtained to date.

## 1. Introduction

Macrocyclic peptides are an appealing chemical class for drug discovery. The structural constraints imbued by their macrocyclic structure provide both an improved target affinity and biological stability relative to their linear counterparts, and many bioactive macrocyclic peptides’ natural products are known. Some of these are clinically useful (e.g., cyclosporine, vancomycin) and others have formed the basis of successful drug discovery programs (e.g., Octreotide development from somatostatin and Caspofungin development from echinocandins). Taken together, this suggests that macrocyclic peptides are a “privileged” chemical class for the discovery of bioactive compounds.

**Figure 1:**
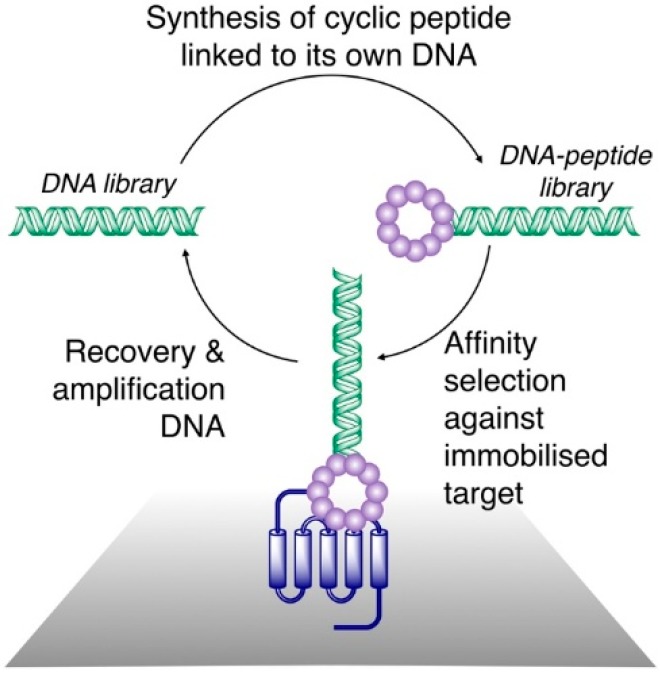
General scheme for affinity selection of macrocyclic peptides from ribosomally synthesized libraries. A DNA library (left) is translated in such a way as to produce a link between each peptide and its cognate DNA, and is cyclized (often through reactions involving Cys residues and leading to formation of thioethers) to produce a DNA–peptide library. This is panned against a surface-immobilized target protein. A DNA library enriched for sequences encoding peptides that bind to the target is recovered and can be used for iterative rounds of selection or can be sequenced to determine the sequences of the peptides with a target affinity.

Over the last decade, a number of biochemical techniques have been developed for the identification of bioactive macrocyclic peptides. While the technical detail of these approaches has been reviewed in detail recently elsewhere, a brief description is warranted here. Such techniques rely on biochemical peptide synthesis (i.e., ribosomal translation) to produce partially randomized peptide libraries that are then cyclized post-translationally, either enzymatically or through non-enzymatic chemical reactions, to yield macrocyclic libraries, which can be screened for target affinity. While these techniques employ diverse methodologies, all of them involve the co-localization of each peptide macrocycle and a cognate encoding nucleic acid. This allows for screening of the pooled libraries, which can be recovered by PCR and deconvoluted by DNA sequencing (Figure 1). This, in turn, allows for the screening of very high diversity libraries, ranging from ~10^6^ (comparable to robotic high-throughput screening methods) to in excess of 10^12^ compounds, depending on the technique used. Consequently, these techniques have become powerful tools for the isolation of macrocyclic peptides with potent activities against targets of interest.

Diverse cyclizing linkages are known in both natural product macrocyclic peptides and in synthetic macrocyclic peptides that are identified using the biochemical screening techniques described above. These include head-to-tail amide bonds, and diverse head-to-side chain, side chain-to-tail, and side chain-to-side chain architectures. Cyclizing moieties involving thioethers are relatively common in both natural product cyclic peptides and synthetic cyclic peptides. In the case of natural products, an entire class of microbially-produced antibiotics, the lantibiotics, are defined by the presence of thioether containing lanthionine moieties, although other non-lantibiotic thioether containing natural products (e.g., thiocoraline) are also known. For biochemical screening approaches, the relatively nucleophilic thiol of cysteine residues can be used as a reactive “handle” for cyclizing chemistries, and the vast majority of macrocyclic compounds identified through such approaches have involved thioether linkages of different types.

The present review focuses on the structural features of target-bound thioether cyclized peptides, identified through biochemical screening approaches (to the best of our knowledge, no structures for thioether bridged natural peptides bound to protein targets have been reported). These are of two general types, (i) bicyclic peptides containing three cysteine residues bridged by reaction with trivalent reagents, and (ii) monocyclic peptides formed either through the reaction of a peptide containing two cysteines with a bivalent linker or through the spontaneous reaction of an N-terminal chloroacetyl group (ClAc) with a downstream cysteine residue (monocyclic thioether containing peptides). These two classes will be discussed in turn.

## 2. Case Studies

### 2.1. Bicyclic Thioether Containing Peptides

**Figure 2:**
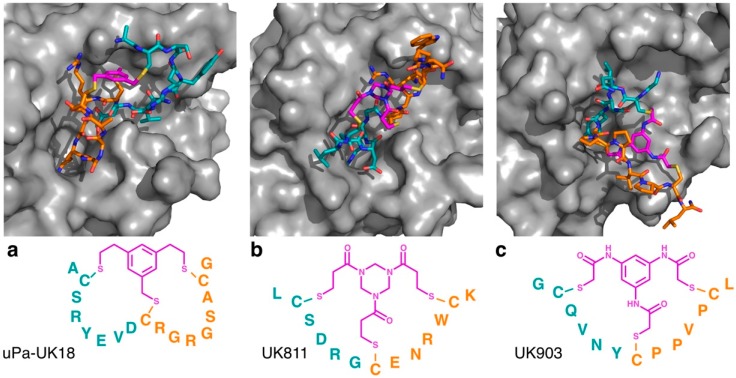
Co-crystal structures of triple thioether closed bicyclic peptides bound to the active site of human urokinase-type plasminogen activator. The 17 residue 1,3,5-tris(bromomethyl)benzene (TBMB) cyclized peptide uPa–UK18 is shown in (**a**) (PDB 3QN7), with the 13 residue 1,3,5-triacryloyl-1,3,5-triazinane (TATA) cyclized peptide UK811 (PDB 4MNX) and *N*,*N*′,*N*′′-(benzene-1,3,5-triyl)tris(2-bromoacetamide) (TBAB) cyclized peptide UK903 (PDB 4MNY) shown in (**b**) and (**c**), respectively. In each case, the trivalent linker moiety is highlighted in magenta, with the first (N-terminal) loop shown in cyan and the second (C-terminal) loop shown in orange.

To the best of our knowledge, only a single protein, the urokinase-type plasminogen activator (uPa), has been crystalized in a complex with thioether cyclized bicyclic peptide ligands, however structures of uPa with several different bicyclic peptide ligands have been described. The first of these to be reported was the structure of the bicyclic peptide uPa–UK18 in complex with uPa [1]. This study found that uPa–UK18 forms an extended structure with no clear classical secondary structural elements (i.e., no helical or sheet elements), but which is nonetheless stabilized by eight intramolecular hydrogen bonds (Figure 2a). Both of the rings of uPa–UK18 formed contacts with uPa, covering an area of approximately 700 Å^2^ and involving 14 intermolecular hydrogen bonds. Subsequent studies showed that the glycine residue at position 13 of uPa–UK18, which exhibited a positive dihedral angle in the crystal structure, could be replaced with d-alanine or d-serine, producing peptides with a slightly increased inhibitory activity and a greatly increased serum stability relative to the parental uPa–UK18 molecules [2].

Notably, however, the cyclizing mesitylene moiety of uPa–UK18 did not interact with the peptide loops, other than through the three covalent linkages (i.e., it did not appear to direct the secondary structure of the scaffold other than through cyclization). To test whether different linkers could alter the secondary structure of bicyclic uPa inhibitors, Heinis and co-workers identified bicyclic peptides based on different trivalent thiol reactive linkers, using an affinity selection approach [3]. These studies found that the use of different linkers dramatically altered the sequences of the peptides identified, and the crystallographic studies demonstrated that these peptides bound to uPa through distinct mechanisms (Figure 2b,c). While most of the linkers used did not form strong non-covalent intramolecular interactions, the TBAB linker (which included the most polar moieties of any of the linkers tested) formed multiple intramolecular hydrogen bonds, suggesting that this linker may be able to directly influence the secondary structure of the peptidic loops. Interestingly, however, the exchange of the linker between the peptides decreased the inhibitory activity of all of the peptides for which this was tested, suggesting that even in the absence of observable hydrogen bonding, small differences in the chemical structure can lead to dramatically different conformations in the constrained peptides.

### 2.2. Monocyclic Thioether Containing Peptides

As a result of the novel techniques for their synthesis and screening, a number of high affinity thioether monocyclic peptide ligands have been identified in recent years, and for several of these, high resolution x-ray crystal structures have been obtained in complex with a protein target. To the best of our knowledge, all of the co-crystal structures of the monocyclic thioether closed peptide ligands reported to date have involved peptides synthesized in genetically reprogrammed reactions, so as to include N-terminal chloroacetyl groups that cyclize with downstream Cys resides. Co-crystal structures of such peptides bound to transmembrane transporters, enzymes, and other proteins (involved in protein-protein interactions) have been reported, and these are discussed in detail below.

#### 2.2.1. Transporters

**Figure 3:**
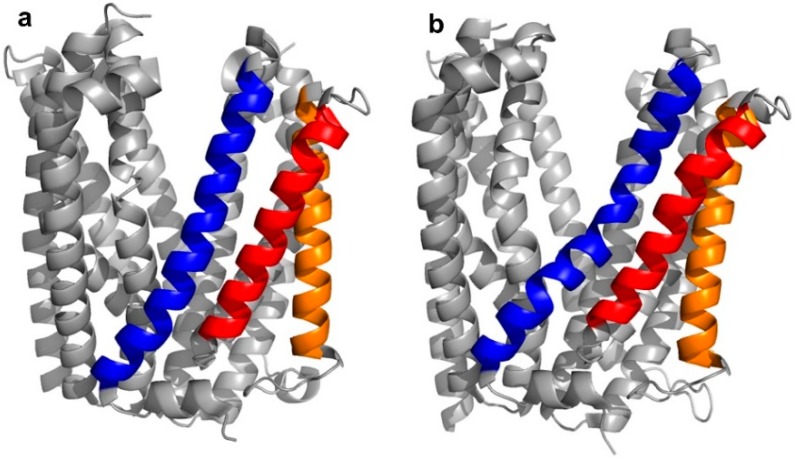
Crystal structures of apo-*Pyrococcus furiosus* multidrug and toxic compound extrusion (PfMATE) in the outwards “straight” (**a**—PDB 3VVN) and "bent” (**b**—PDB 3VVO) conformations. The transmembrane (TM) domains that are most affected by the structural transition are colored in blue (TM1), red (TM5), and orange (TM6). Among these, TM1 shows the most apparent structural change, with the “bent” conformation named after the bending of its central motif.

In the case of the transmembrane transport proteins, thioether cyclized peptide ligands have been used specifically as co-crystallization ligands in order to facilitate the crystallographic analysis of these relatively intractable proteins. In the first example of this, high affinity macrocyclic peptide ligands were identified to the *Pyrococcus furiosus* multidrug and toxic compound extrusion (PfMATE) transporter, a representative member of a diverse family of xenobiotic efflux proteins that confer multidrug resistance to microbial pathogens and neoplastic cells [4,5]. Structurally, the transporter was found to adopt two different “straight” or “bent” conformations, according to the spatial arrangement of its transmembrane (TM) domains, in particular, TM1, TM5, and TM6 (Figure 3a,b).

**Figure 4:**
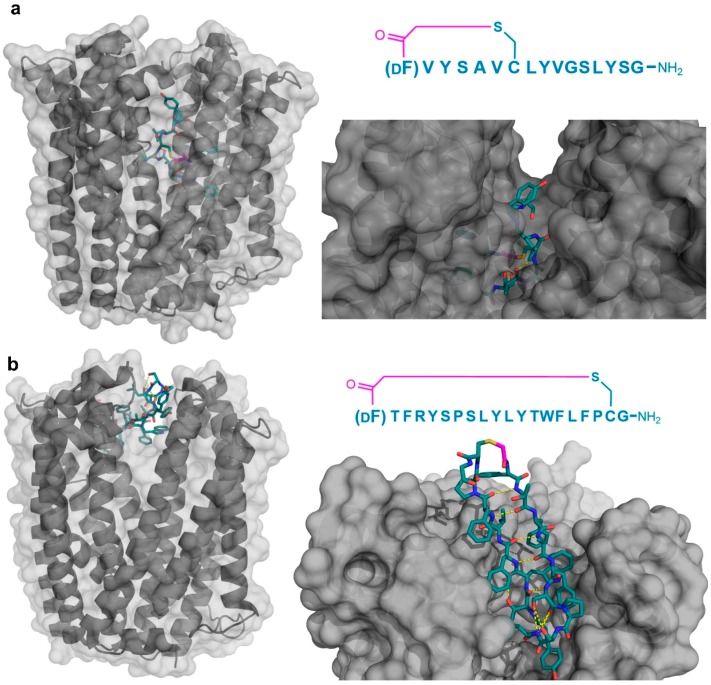
Co-crystal structures of pfMATE transporter (gray) in complex with the thioether cyclized peptides MaD3S (**a**—PDB 3VVS) and MaL6 (**b**—PDB 3WBN). The overall location of the peptide in the transporter is shown on the left, while an upper magnified view (darkened) on the right. Intramolecular polar interactions of the peptides are shown in yellow. The thioether cyclizing linker is highlighted in magenta. DF—d-phenylalanine.

Four peptide ligands to PfMATE were identified (MaD5, MaD3S, MaD8, and MaL6), and each of these exhibited a strong inhibition of PfMATE extrusion, suggesting their possible use as inhibitors as well as co-crystalization ligands. Three of these (MaD5, MaD3S, and MaD8) included a single d-Phe residue and exhibited “lariat” structures with relatively small N-terminal macrocyclic regions (5–7 residues) and longer C-terminal tails (9–13 residues). By contrast, in the MaL6 peptide (which did not include any d- residues), all 17 residues were included in the macrocyclic structure. The co-crystal structures of these peptides bound to PfMATE showed that MaD5 and MaD3S bound within a deep central cleft pocket of a straight conformation-locked PfMATE, with the macrocyclic region of each peptide occupying a substrate recognition site (the N-lobe cavity). The interaction of the macrocyclic domains with PfMATE was mainly mediated through hydrophobic interactions, with the C-terminal tails adopting disordered positions that were not completely defined (Figure 4a). In contrast, MaL6 and MaD8 were shown to bind to the extracellular opening of the TM domains, with MaD8 binding to a site deep within the channel, and MaL6 binding to the outward face of the transporter (Figure 4b). The distinct binding mode of each peptide suggested distinct mechanisms of inhibition, with the long tails of the lariat, for example, appearing to restrict the motion of the N- and C-terminal lobes necessary for conformational changes during extrusion. Additionally, the peptides were found to reach the transporter intracellularly after penetrating the bacterial membrane, proceeding to inhibit the transporter’s function through spatial blocking or through restriction of the TM’s dynamic movement. Overall, these studies allowed for the identification of cyclic peptides that both inhibited PfMATE and facilitated its crystallization, providing insight into both the modes of macrocyclic peptide binding and inhibition, and the mechanism of the transporter’s activity.

**Figure 5:**
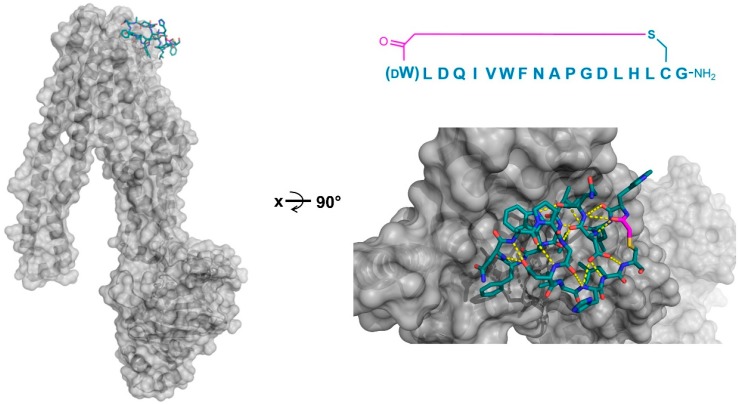
Co-crystal structures of the P-glycoprotein homolog and ABC multidrug transporter CmABCB1 (gray) in complex with its selected macrocyclic inhibitor, aCAP (PDB 3WMG). Intramolecular polar interactions of the peptide are shown in yellow. The thioether cyclizing linker is highlighted in magenta. DW—d-tryptophan.

In addition to PfMATE, a different high affinity thioether cyclized peptide ligand was identified against another xenobiotic transporter, the CmABCB1 protein from the red alga Cyanidioschyzon merolae [6]. In this case, the macrocyclic ligand, aCAP, was an 18 amino acid thioether-cyclized macrocyclic peptide, which, like the PfMATE ligands described above, also functioned as a CmABCB1 inhibitor. The co-crystal structure obtained comprised an inward-open conformation for CmABCB1, with aCAP bound to the extracellular surface (Figure 5). The peptide was found to interact with a “gate” in the extracellular region of the protein formed by tightly packed TMs, acting as a clamp that restrained their conformation. From the additional mutational and transport studies, a full scheme of the transport mechanism was elucidated. It was found that the extracellular gate in the upper side of the complex is maintained by strong interactions between the TM domains, particularly TM1 and TM6. Upon the binding of the substrate in the cavity of the transporter, it interacts with a Tyr residue in TM5 that promotes the movement of the upper domains in opposite directions, consequently disrupting the interactions of the extracellular gate and generating its opening, while also accelerating the ATPase activity. However, upon the binding of aCAP, the interactions between the TMs in the extracellular gate are reinforced, preventing the opening of the gate and efficiently inhibiting the transport activity.

#### 2.2.2. Enzymes

**Figure 6:**
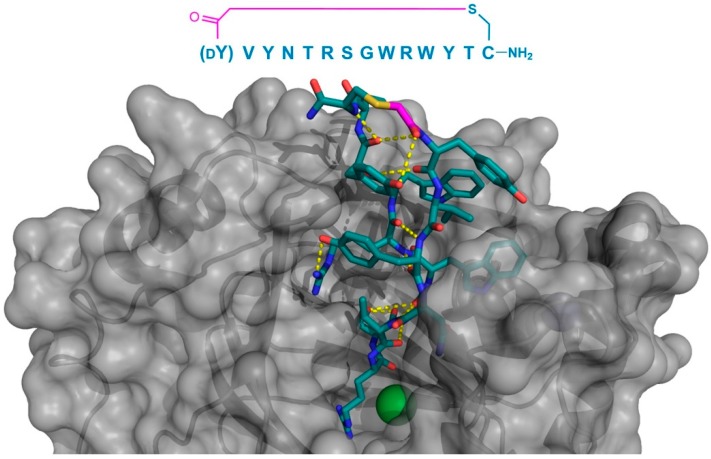
Co-crystal structure of JmjC-domain Lysine Demethylase KDM4A (gray) in complex with the thioether cyclized peptide CP2 (PDB 5LY1), a selected macrocyclic peptide inhibitor (the inhibitor’s sequence is shown above the structure). Intramolecular polar interactions in CP2 are shown as yellow dashed lines, Zn^2+^ ion as a teal sphere, and Ni^2+^ (replacing Fe^2+^ in KDM4A crystals) as a green sphere. The thioether cyclizing linker is highlighted in magenta. DY—d-tyrosine.

In addition to the use of thioether-cyclized macrocyclic peptides as inhibitors/co-crystallization ligands of membrane transporters, compounds of this class have been identified as inhibitors of specific enzymes [7,8,9,10], and in several cases, these have been subjected to X-ray crystallography as a basis for the subsequent structure–activity studies. For example, Kawamura et al. initially identified several peptides with strong binding and inhibition properties against the JmjC-domain containing lysine demethylases (JmjC-KDMs), a family of Fe^2+^ and 2-oxoglutarate dependent histone modification enzymes [11]. In particular, the macrocyclic peptide CP2 was found to exhibit the potent inhibition of the KDM4A and KDM4C, and was crystallized bound to KDM4A. This peptide demonstrated a surprising binding mode, localized not to the catalytic 2OG-binding pocket, but to the histone-binding domain, where it formed a two-turn β-sheet stabilized by, and interacting with KDM4A through multiple hydrogen bonds. The Arg6 of CP2 was found to bind to the sub-pocket usually occupied by trimethyl Lys in the histone substrate, mimicking its positive charge, and localized near the protein’s Fe^2+^ cofactor (Figure 6). Based on this co-crystal structure, a number of alterations were made to CP2 (e.g., the N-methylation of specific backbone positions or their substitution with a d- or fluorinated-amino acids), with the aim of improving the stability and cellular uptake. Most of these modifications were well-tolerated, even when combined, and compounds with an improved activity in cell culture assays were obtained, demonstrating the potential for the structure-based design of macrocyclic peptides.

**Figure 7:**
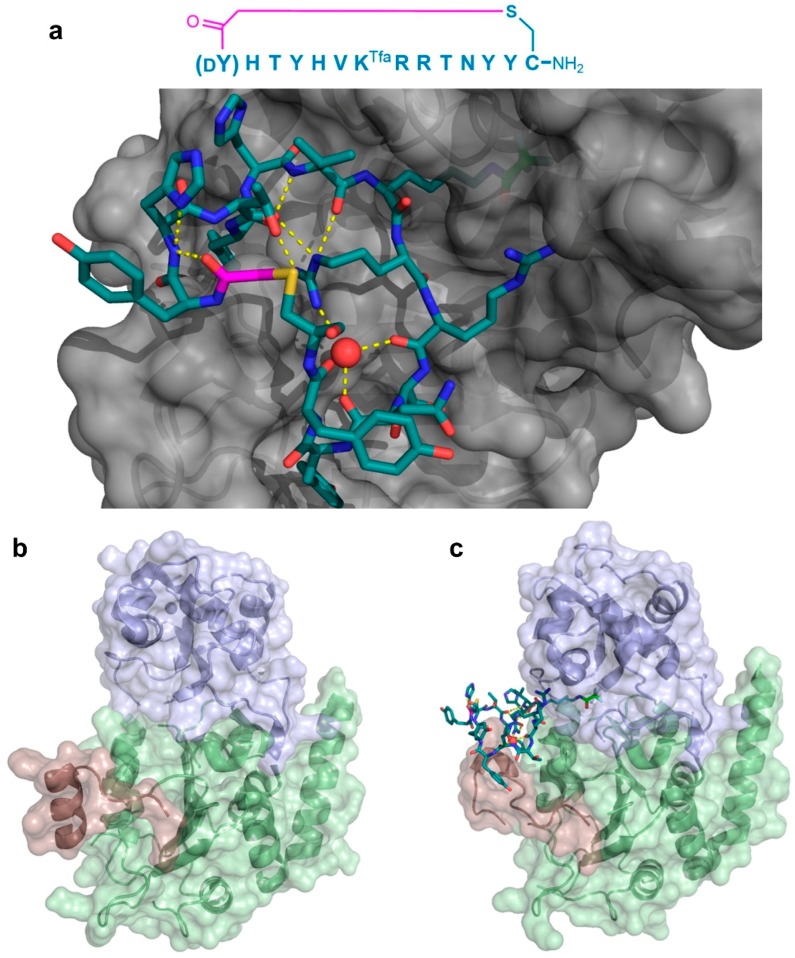
Co-crystal structure of the Sirtuin 2 (SIRT2) first unit in complex with the S2iL5 macrocyclic peptide inhibitor. The (**a**—PDB 4L3O) sequence of the inhibitor and its binding site on the groove between the small and large domains of the enzyme (gray) are shown. The peptide maintains a highly rigid structure with several hydrogen bonding interactions (yellow dashed lines), including ones mediated by a water molecule (red sphere). The K^Tfa^ warhead is highlighted in light green. Below, the significant structural changes in the SIRT2-specficic region (red) upon binding of the peptide between the small (blue) and large (green) domains are apparent when comparing the free enzyme (**b**—PDB 1J8F) and its bound form (**c**). The Zn^2+^ cofactor in the small domain is shown as a gray sphere, and the thioether cyclizing linker is highlighted in magenta.

As a second example of the X-ray crystallographic analysis of a thioether-cyclic peptide targeting an enzyme, Yamagata et al. analyzed the structure of the S2iL5 macrocyclic peptide bound to its target, the human NAD^+^-dependent deacetylase Sirtuin 2 (SIRT2) [12,13]. S2iL5 was originally isolated from a peptide library constructed around a trifluoroacetyl Lys residue (K^Tfa^), designed to act as a mechanism-based “warhead” targeted to the SIRT2 active site. The co-crystal structure of S2iL5 and SIRT2 demonstrated that S2iL5 assumed a remarkable structure, in which the cyclic peptide scaffold was stabilized by a central water molecule, effectively coordinated by Arg8, Arg9, and Asn11 (Figure 7a). This structure allowed for the peptide to bind to a groove of SIRT2, presenting the warhead directly into the active site. Surprisingly, not only did the peptide and protein engage in a β-sheet-like mode of interaction that facilitated the transition of SIRT2 to a more closed state, but the analysis of the unbound SIRT2 showed that the SIRT2-specific insertion region (residues 289–304) went through a drastic structural change from a full α-helix to a loop conformation upon peptide binding (Figure 7b,c). The subsequent mutational studies on both the peptide and the protein revealed that this region is remarkably flexible and may play an important role in the substrate recognition, with each interaction with the peptide contributing synergistically to the overall kinetics of the conformational change. Furthermore, these studies indicated that this large structural change was facilitated by the macrocyclic skeleton of the peptide, through its structural plasticity and numerous interactions with the target.

**Figure 8:**
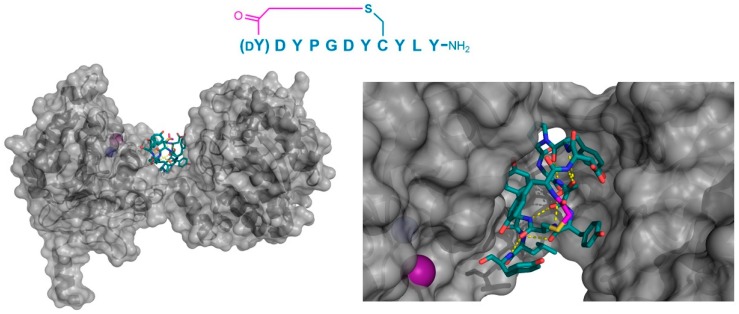
Co-crystal structure of *C. elegans* iPGM (gray) in complex with the peptide inhibitor, Ce-2d (PDB 5KGN). The left panel shows the positioning of the tightly folded peptide in the groove between the phosphatase and transferase domains of the protein. On the right, a magnified view shows the multiple Tyr residues in the peptide, as well as the intramolecular polar interactions (yellow dashed lines) and the presence of the N-terminal Tyr11 amide’s oxygen in close proximity to the Zn^2+^ cofactor (purple sphere). The Mn^2+^ (blue sphere) cofactor is shown as a blue sphere, and the thioether cyclizing moiety is highlighted in magenta.

In a third example, the co-crystal structure of a thioether-cyclized macrocycle inhibitor (Ce-2d) of the *Caenorhabditis elegans* co-factor independent phosphoglycerate mutase enzyme (iPGM) was determined [14]. Similar to the MaD5, MaD3S, and MaD8 peptides described above, Ce-2d exhibited a “lariat” structure with a d-Tyr initiated, thioether-closed macrocycle of eight residues, followed by a linear “tail” of three amino acids. The crystal structure of Ce-2d with the *C. elegans* iPGM demonstrated that the macrocycle of Ce-2d was bound between the phosphatase and transferase domains of iPGM in a dominantly polar cavity, leaving the four amino acid tail free to form a small α-helical structure in contact with the solvent, with Tyr11 in close proximity to the Zn^2+^ and Mn^2+^ ion cofactors (Figure 8). The superposition of this structure with the 2-phosphoglycerate substrate showed that Ce-2d did not interact directly with either the substrate or the active site, evidencing an allosteric effect. Studies of the truncated variants of Ce-2d demonstrated that the terminal Tyr 11 was critical for activity, probably because of the hydrogen bonding of the carboxamide of this residue with iPGM Glu87. These studies also identified a more potent analogue of Ce-2d, Ce-2a, which exhibited the sub-nanomolar inhibition of iPGM, but for which the co-crystal structure could not be determined. Ce-2a includes a longer (seven residue) “tail” than Ce-2d and a terminal Cys residue. The modeling of this analog suggested an interaction of this terminal Cys with the Zn^2+^ co-factor of iPGM, explaining the considerably stronger inhibitory activity of Ce-2a compared with Ce-2d. Overall, these thioether-closed macrocycles were found to induce allosteric inhibition, stabilizing a locked-open structure of iPGM, and, in the case of Ce-2a, sequestering its Zn^2+^ co-factor.

**Figure 9:**
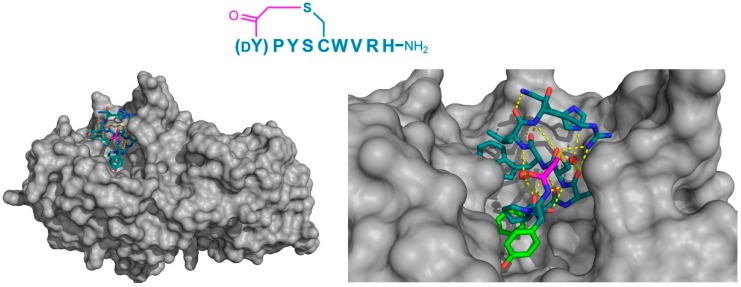
Crystal structure of HPA (gray) in complex with the macrocyclic peptide inhibitor, piHA-Dm (PDB 5KEZ). The left-hand panel shows the position of macrocyclic peptide binding in the active site of the enzyme. On the right, a magnified view shows the intramolecular hydrogen bonding interactions (yellow dashed lines) within the peptide, including those with two water molecules (red spheres). The dY-P-Y motif has been identified as playing a crucial role in inhibition, and is highlighted in green. The thioether cyclizing linker is highlighted in magenta.

Finally, the thioether cyclized peptide ligands have also been co-crystalized bound to the human pancreatic amylase (HPA), an enzyme involved in starch digestion and implicated as playing a role in type-2 diabetes [15]. These peptides exhibited conserved RFGYAY and (dY)PYSCWXRH motifs, and a lariat architecture, and were potent competitive inhibitors of HPA, with inhibition constants in the single digit nanomolar range. The co-crystal structure of one of these peptides, a nonapeptide termed piHA-Dm, showed that it occupied the catalytic site of the protein, consistent with its competitive mechanism. The tail region of the lariat assumed a highly ordered α-helical structure, while the five-membered cycle was localized and tightly compacted within the catalytic site and bound directly (or through water molecules) to several key catalytic amino acids in the protein (Figure 9). Furthermore, even the initiating d-Tyr was shown to form important interactions with the protein, through both its side chain and amide moieties. The d-stereochemistry of the initiating Tyr was found to allow a parallel arrangement with Tyr3, forming a tripeptide motif with Pro2 that made crucial interactions with the protein that were strikingly similar to the known inhibitors of HPA. On the basis of these findings, the authors hypothesized that the (dY)PY and YAY motifs may be responsible for the inhibitory activity observed in all of the peptides identified, and that similar two-phenolic moiety motifs may have the potential for the development of inhibitors targeting amylases more generally.

#### 2.2.3. Other Targets

**Figure 10:**
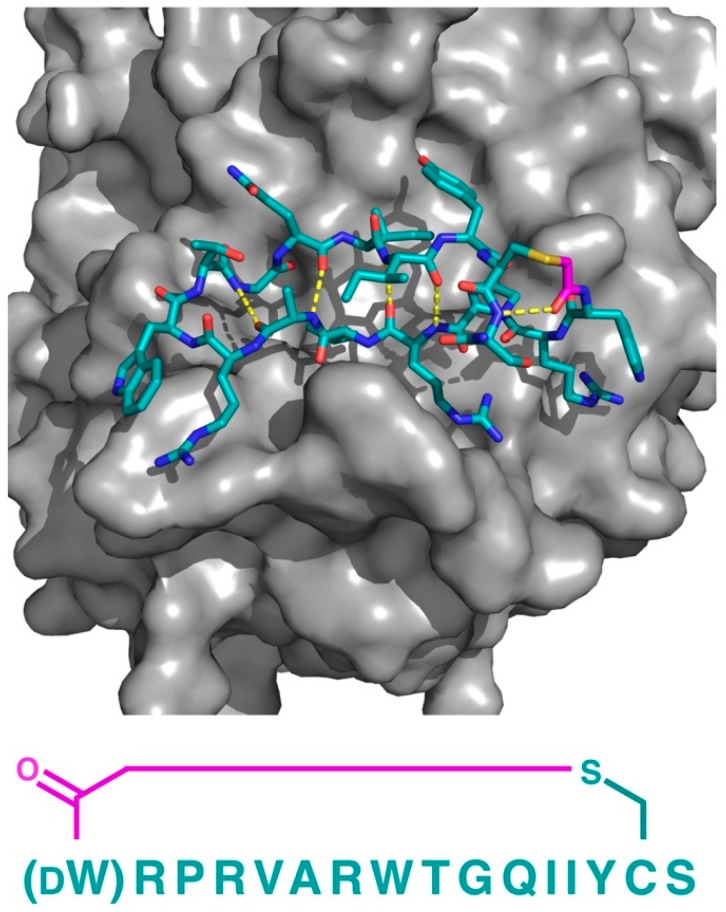
Co-crystal structure of the thioether closed cyclic peptide PB1m6 bound to human Plexin B1 (PDB 5B4W). Hydrogen bonding between the backbone amides (dashed yellow lines) stabilizes the antiparallel β-sheet like structure of PB1m6. The sequence of the peptide (left-to-right, N-to-C) is also shown. The thioether linker moiety is highlighted in magenta.

In contrast to traditional small molecules, cyclic and bicyclic peptide ligands can be identified against essentially any protein target, and are not limited to targets with appropriate binding pockets, such as transporters and enzymes, making them amenable to the targeting of protein–protein interactions. However, to the best of our knowledge, only a single co-crystal structure for a thioether containing peptide macrocycle protein–protein interaction inhibitor (not including the KDM4 and SIRT2 inhibitors described above, which, while technically involved in protein–protein interactions, involve enzymatic processes) has been reported. In this study, Matsunaga and co-workers identified a high affinity (*K*_D_ = 3.5 nM) thioether-closed cyclic peptide ligand to Plexin B1 (termed PB1m6), which was also a potent inhibitor of the interaction of Plexin B1 with Semaphorin 4D; an interaction that regulates osteoblast differentiation and is a possible target for osteoporosis [16]. Remarkably, the co-crystal structure of Plexin B1 with PB1m6 demonstrated that the peptide ligand bound at a site significantly distant from the Semaphorin interacting region and was an allosteric inhibitor (Figure 10). Interestingly, the bound PB1m6 formed a short section of anti-parallel β-sheet, stabilized by four backbone amide hydrogen bonds and Arg–Trp cation–pi interactions at the two turns, demonstrating the capacity of relatively short thioether cyclized peptides to form recognizable secondary structure motifs.

## 3. Conclusions

The very high affinity and selectivity of thioether-closed macrocyclic peptides, as well as the relative ease with which they can be identified using modern techniques, make them intriguing ligands for diverse applications. As described above, such compounds can form highly diverse structures because of their ability to adopt different spatial conformations, and sections of recognizable protein secondary structure (α-helices and β-sheets) can be observed. Unlike smaller molecules, the bound structures of macrocyclic peptide ligands appear to always involve intramolecular interactions in the macrocycle, which presumably stabilize their binding conformations and allow them to adopt the requisite conformations for binding to highly diverse “pockets”. At present, the relative paucity of structural information (only a few co-crystal structures are currently available) makes it difficult to draw further general conclusions about the binding modes of thioether-closed macrocyclic peptides to their targets. However, the utility of such compounds, and the fact that their rate of discovery is increasing year by year, leads us to believe that many more co-crystal structures will be solved in the near future, allowing for further insights into the structural biology of these particularly interesting compounds.
